# Double-Edged Sword Effect of Information Usage Transparency in Privacy Policies on Users’ Continuous Use Intention of Apps

**DOI:** 10.3390/bs16081302

**Published:** 2026-07-31

**Authors:** Jingyun Wang, Wenxia Liu, Guanfeng Shi

**Affiliations:** 1 School of Economics and Management, Shihezi University, Shihezi 832003, China; 2 Department of Corporate Governance and Management Innovation Research Center, Shihezi University, Shihezi 832003, China

**Keywords:** information usage transparency, privacy policy, perceived privacy fatigue, perceived privacy control, mobile commerce

## Abstract

The mobile commerce era witnesses widespread data collection by various apps. These data practices optimize service experience. They also trigger broad public concerns regarding privacy leakage. Transparency about information use in privacy policies is critical. It balances data utilization and privacy protection. In practice, highly transparent information-use provisions create a double-edged sword effect on users. Different individuals hold different perceptions of such transparency. This study relies on Stress and Coping Theory. It analyzes two dual mechanisms: benefit-enhancing and detriment-weakening. Information usage transparency affects users’ continuance intention to use the app through these two mechanisms. The study also explores the boundary conditions of the mechanisms. Three scenario-based experiments collect valid data. The data test the proposed theoretical model. Results indicate that: (1) Information usage transparency significantly boosts users’ app continuance intention via perceived privacy control. Information usage transparency also significantly reduces such continuance intention via perceived privacy fatigue. (2) Text information sensitivity strengthens the benefit-enhancing mechanism. It weakens the detriment-weakening mechanism. This study clarifies how information usage transparency shapes users’ continuance intention. It provides practical guidance for app service providers. Providers can optimize privacy policy design with this guidance. They can effectively improve user stickiness.

## 1. Introduction

Data is a core strategic asset for enterprises to build competitive advantage. The digital economy is developing rapidly. Data grows increasingly important in this context (Cai et al., 2022). App service providers operate within online privacy environments. They leverage user data via precise recommendation mechanisms. These mechanisms match supply and demand efficiently. They also raise user utility (Pang et al., 2020). Users gain higher utility from such services. However, data privacy risks accompany data use. Identity information leakage is one typical risk. These risks continually deepen users’ concerns about personal privacy (Xu et al., 2022; Cohen, 2018). Global privacy supervision rules are evolving rapidly to address the above concerns. The EU enforces the General Data Protection Regulation (GDPR) and the Digital Markets Act (DMA). The United States is rolling out comprehensive state-level privacy laws, such as the California Privacy Rights Act (CPRA). These laws set a new industry standard. Transparency is no longer optional. These regulatory rules require all organizations to clearly inform users about the purposes, scope, and modes of data use. Two conflicting demands appear after the release of these rules. Enterprises hope to mine data value. Users need full privacy rights protection. This conflict becomes a core problem in digital technology governance. Drawing up privacy policies is an important measure for app service providers. It protects users’ personal information. It also serves as a vital channel for building user trust. But privacy policies bring contradictory practical results. On the one hand, detailed disclosure of information-use rules enhances transparency in information use. It helps enterprises build user trust (Esteves et al., 2022). On the other hand, long and complicated policy clauses greatly raise users’ cognitive burden and mental pressure. App service providers operate in online scenarios. They cannot rely only on optimizing technical functions to retain users. Enterprises need to create a scientific design paradigm. This paradigm can ease users’ privacy worries and cut cognitive friction. It is the only effective way to increase users’ intention to continue use.

Scholars explore how privacy policies affect user behavior. Most existing research examines external features of privacy policies. These features include policy existence (Obar & Oeldorf-Hirsch, 2020), readability (Guo et al., 2022), and structural layout (Shezan et al., 2022; Kitkowska et al., 2023; Alfawzan et al., 2022). These studies mainly discuss how such features encourage users to adopt apps for the first time or share personal information. Scholars pay limited attention to one key mechanism. The core factor here is information usage transparency. It is a core content feature. It decides whether privacy policies work effectively. Few studies explain how information usage transparency generates impacts. Some research verifies that transparent privacy policies can strengthen user trust (Martin et al., 2017). Other studies hold different views. They point out that transparent policy content may cause information overload (Awad & Krishnan, 2006). Conflicting research results appear for one major reason. Past research has ignored the complex psychological processes arising from information usage transparency. Most prior studies treat the practical effect of privacy policies as a one-dimensional concept. They mainly test linear relationships between policy features and user responses (Resnick & Montania, 2003). These studies overlook two key points. First, information usage transparency may have a double-edged effect on user behavior via separate mediating paths. Second, they fail to explain how different types of information set boundary conditions for such effects. This study takes Stress and Coping Theory as its theoretical foundation. It centers on information-use transparency in privacy policies. It analyzes two separate psychological paths. These paths explain how information-use transparency shapes users’ intention to continue use. Text information sensitivity is added to the research model as a moderating variable. This setting can systematically identify boundary conditions between core variables. This research perspective delivers two values in academia and practice. First, it resolves inconsistent theoretical conclusions. It deepens scholars’ comprehension of the practical effects of privacy policies. Second, it offers targeted practical suggestions for app service providers. Providers can design clear, transparent, and user-friendly privacy policies with these references.

## 2. The Relevant Literature

### 2.1. Information Usage Transparency

Privacy policies act as an industry self-regulatory tool. The tool restrains internet companies from misusing user information. It also lowers information security risks. Privacy policies list relevant rules clearly. They tell users how enterprises collect, use, and protect personal information. Scholars research privacy policies. Their research focus shifts from the mere existence of privacy policies to their quality. Early research mainly analyzes whether privacy policies exist. It tests how this existence affects user trust. More recent studies grow in quantity. These studies examine external display features of privacy policies. They discuss how such features shape user experience (Guo et al., 2022; Shezan et al., 2022; Kitkowska et al., 2023; Alfawzan et al., 2022). Information usage transparency is a core content dimension of privacy policies. It describes how clear the core usage rules are in privacy policies. These rules cover information collection purposes, usage scenarios, third-party sharing parties, and data storage periods. Users rely on information-use transparency to assess their own information security level (Martin et al., 2017). Mobile apps usually pop up privacy policy windows in the background. The pop-ups appear when users open or run the app for the first time. This step actively releases relevant information via a signal-transmission mechanism. This mechanism forms privacy policies with high transparency in information use. It narrows the information gap between enterprises and users. Thus, privacy policies become an important medium for delivering information about usage transparency (Awad & Krishnan, 2006).

Scholars hold conflicting opinions on the effectiveness of information usage transparency. Most scholars recognize the value of information usage transparency. They state that clear privacy policies with sufficient information usage transparency boost credibility. Such privacy policies also have positive effects on the user experience (Resnick & Montania, 2003). Information usage transparency relieves information imbalance between enterprises and users. It helps enterprises build stable user relationships (Martin et al., 2017). Other studies identify adverse outcomes brought by excessive information usage transparency. Transparent data disclosure lets users gain a sense of control over their personal data. However, overly detailed disclosure content requires users to read large volumes of text. This task creates constant mental strain. It eventually leads to perceived privacy fatigue. Srinath et al. built the PrivaSeer corpus. The corpus can analyze complicated privacy policies. The research goal is to standardize and simplify these privacy policies. The simplified versions match users’ practical demands (Srinath et al., 2021). Research directions have changed over time. This trend indicates a key possibility. Users may refuse to read privacy policies. The detailed and complex content imposes a heavy cognitive burden on users. This outcome reduces the real practical value of privacy policies. Different research contexts lead to inconsistent research conclusions. This factor explains the divergence of existing study results.

Conflicting research results form the current research backdrop. Scholars engage in deeper discussions against this backdrop. The discussions focus on the contextual boundary conditions of the effects of information usage transparency. Scholars analyze the evolution of security and privacy research. Information-use transparency is a vital prerequisite. It supports effective intervention targeting users (Reuter et al., 2022). Usage scenarios feature high diversity. Customized schemes are needed to resolve this challenge. Existing research findings reflect a clear shift in transparency-related research. Early studies discuss whether information usage transparency should be adopted. Newer studies launch refined discussions. The discussions focus on two core questions: how to implement information-use transparency and what degree of transparency is appropriate. The above research trend exists within mobile commerce scenarios. App service providers need to balance two key points. They need to deliver sufficient information notifications to users. They also need to avoid triggering information overload. Finding this balance has become an urgent issue. The issue matters for both theoretical research and practical operation.

### 2.2. Perceived Privacy Control and Perceived Privacy Fatigue

Perceived privacy control refers to an individual’s perceived degree of control. The control targets the processing of their personal information on the service side. It stands for users’ ability to set privacy boundaries. It also relies heavily on specific contexts. External environmental variables can easily adjust their effect (Mutimukwe et al., 2020). This psychological construct affects people’s privacy behaviors and attitudes across a range of scenarios. Its function is vital. Greater perceived privacy control builds user trust in online environments. It also lowers users’ privacy worries (H. Li et al., 2014). Bartol et al. carried out relevant research. Their findings show that a strong perceived privacy control reduces privacy fears. It also promotes online activities based on network technologies (Bartol et al., 2024). Chang et al. put forward the privacy boundary management model. The model explains a clear mechanism. Users set and manage privacy boundaries actively according to their perceived privacy control. This state further changes users’ privacy behaviors in online banking scenarios (Chang et al., 2018). Markos et al. obtained another research conclusion. Perceived privacy control changes people’s willingness to share personal data. Such influences vary by the type of disclosed information (Markos et al., 2017).

The concept of perceived privacy fatigue emerges in the internet era. Privacy risks and flaws in personal information security are becoming increasingly serious during this period. Acquisti proposed a definition of perceived privacy fatigue. It refers to users’ tired feelings. These feelings stem from unexpected data leaks and complicated online perceived privacy control. The research also verifies that this psychological state reduces users’ focus on privacy matters (Acquisti et al., 2006). This study defines perceived privacy fatigue on its own terms. It describes users’ bored, exhausted mental states when they read lengthy privacy policies. Existing research shows that users’ emotional states affect their intention to continue use (K. Zhang et al., 2025). Perceived privacy fatigue is one typical emotional state. It influences users’ behavioral intentions. Qiu et al. provided relevant empirical evidence. Perceived privacy fatigue drives users to switch between health information platforms. This effect becomes more pronounced when users perceive privacy protection as inadequate (Qiu et al., 2026). Users with strong privacy worries are more likely to generate cognitive fatigue when handling privacy-related matters. Li et al. pointed out that perceived privacy fatigue can cut down users’ active participation. It also makes users take more passive actions (J. Li et al., 2022). Zhu et al. combined privacy calculus and perceived privacy fatigue in their research. The research explains the formation of the privacy paradox. The paradox means that users still share personal information despite privacy concerns. The study emphasizes that perceived privacy fatigue is a key factor driving such contradictory behavior (Zhu et al., 2021). All the above studies draw a unified conclusion. Perceived privacy control and perceived privacy fatigue are two core variables. They affect users’ privacy worries, information disclosure behaviors, and privacy management strategies in all crowds and scenarios. The two variables can greatly shape users’ attitudes, emotions, and behavioral intentions. The result underscores the need to develop privacy protection technologies and privacy policies. Such technologies and policies need to match users’ perceptions and expectations.

### 2.3. Text Information Sensitivity

Users do not hold fixed attitudes toward privacy issues. Their responses vary greatly depending on the context. The type of information involved acts as a key contextual factor. Petronio proposed the communication privacy management theory. The theory holds that people set privacy boundaries for their private information. Multiple factors decide how easily these boundaries can be crossed. Such factors include the type of information and cultural background (Petronio, 2002). The research background falls within mobile commerce. Different types of personal information carry varying levels of privacy risk and value. This difference is defined as information sensitivity. Information sensitivity refers to the potential risks associated with personal information. Different types of information have distinct risk characteristics (Kitkowska et al., 2020). Text information sensitivity refers to risk attributes covered in the privacy policy text. This is the definition adopted by this study. Many existing studies point out a consistent classification standard. The public regards financial and identity data as highly sensitive. Purchasing records and lifestyle data are considered low-sensitivity information (Malhotra et al., 2004). This classification difference directly changes users’ choices of information disclosure. Users are willing to share low-sensitivity information. They take a cautious approach to highly sensitive information. They may generate negative feelings about the collection of highly sensitive information. Some users even refuse authorization for relevant data. Most previous studies on the effects of privacy policies treat all information involved as identical. Some studies only control one type of information. They do not take text information sensitivity as an independent moderating variable. The variable is not systematically added to the influence model of information usage transparency. The inclusion of text information sensitivity can clarify reasonable adjustment methods of information usage transparency. Such adjustments can amplify the positive effects brought about by privacy policies. It also delivers an accurate theoretical basis for enterprises. Enterprises can design differentiated and refined privacy policies based on such conclusions.

## 3. Research Hypotheses and Theoretical Model

### 3.1. The Mediating Role of Perceived Privacy Fatigue

The online privacy environment features complicated privacy protection agreements (Shao et al., 2022). Platforms also frequently ask for personal information at varying levels of detail. These two factors raise users’ reading workload. They create psychological pressure and fatigue (Choi et al., 2018). Every person has an internal limit for external privacy interactions. Outside privacy demands go beyond this limit. Users will feel exhausted by daily privacy management work. Stress and Coping Theory offers relevant theoretical logic. People will judge potential opportunities and threats from stressors when they sense pressure (Lazarus, 1984). Information-use transparency serves as an external stimulus. Its completeness and clarity guide users’ cognitive judgment and shape final evaluation results. Users expend more energy reading privacy policy content when it is more detailed. Rising cognitive load leads users to treat the stimulus as a threat. This process generates perceived privacy fatigue (Han et al., 2026). Service providers provide thorough descriptions of how information is used. Such detailed disclosures enable users to recognize more risks in the stimulus. This situation worsens perceived privacy fatigue. Based on the above theoretical and empirical framework, the following hypothesis was developed:

**Hypothesis** **1.** *High (vs. low) information-use transparency induces greater perceived privacy fatigue.*

Fatigue mainly comes from conflicts between high demands and unattainable goals. Many existing studies discuss the behavioral outcomes of perceived privacy fatigue. These studies prove that perceived privacy fatigue greatly affects people’s privacy decisions. It usually weakens users’ behavioral performance. Dhir et al. carried out targeted research. Their results show that perceived privacy fatigue lowers users’ attention and participation efficiency (Dhir et al., 2019). Keith et al. put forward another discovery. Harder access to perceived privacy control makes users refuse to handle privacy-related choices. This finding indirectly demonstrates that perceived privacy fatigue strongly influences users’ information behaviors (Keith et al., 2014). Stress and Coping Theory presents additional perspectives. People under fatigue may withdraw from ongoing tasks. They will take behavioral disengagement strategies. These strategies mean people actively cut down efforts to handle stressors. Some people even stop responding to relevant disturbances. Based on the above theoretical and empirical framework, the following hypothesis was developed:

**Hypothesis** **2.** *Perceived privacy fatigue significantly negatively impacts users’ intention to continue using the app.*

A core goal of privacy research is to explore how users’ perceptions of information usage transparency affect their sustained engagement (Dehling & Sunyaev, 2024). In the online privacy context, information usage transparency is a key factor. This factor shapes users’ online privacy behaviors. Excessive information usage transparency may place heavy decision-making burdens on users. It weakens users’ information-processing capacity (Peng et al., 2021). Online platforms may provide overly complex privacy management options. These options create barriers to users’ understanding of their privacy preference settings (Wang & Li, 2025). Massive information processing consumes users’ mental energy. This process triggers psychological fatigue. This fatigue originates from psychological pressure. It further lowers users’ work efficiency and decision-making ability. It may finally cause users to abandon the app. Based on the above theoretical and empirical framework, the following hypothesis was developed:

**Hypothesis** **3.** *Information usage transparency negatively influences users’ continuous use intention for apps through the mediating role of perceived privacy fatigue.*

### 3.2. The Mediating Role of Perceived Privacy Control

Stress and Coping Theory explains how people respond to stressors in their environment. People judge perceived benefits when facing stressors (Lazarus, 1984). Perceived privacy control is a type of positive control in online privacy scenarios. It usually brings higher perceived value than potential economic gains. Platforms interact with users. The platforms raise transparency in information use and autonomy in information use. The measures give users real control over their personal information. This method helps form long-term willingness to cooperate. Highly transparent privacy policies will not weaken user trust. Such privacy policies clearly deliver users’ data control rights. They strengthen user trust. This effect supports stable long-term user relationships (Veltri et al., 2023). Information usage transparency has two opposing effects. Users form two kinds of perceptions about this transparency. These perceptions lift users’ perceived privacy control. Based on the above theoretical and empirical framework, the following hypothesis was developed:

**Hypothesis** **4.** *High (vs. low) information usage transparency enhances perceived privacy control.*

Weak perceived privacy control over personal information serves as a major trigger of user privacy anxiety. Higher perceived privacy control over personal information boosts users’ long-term willingness to cooperate with service providers (Rahman et al., 2019). Service providers collect and store users’ personal information in online privacy environments. Users can access online services at any time and from any place. Users hold a stronger sense of information control. They become more accepting of service providers’ utilization of personal information. Such information covers sensitive data (Kim et al., 2019). Based on the above theoretical and empirical framework, the following hypothesis was developed:

**Hypothesis** **5.** *Perceived privacy control significantly positively impacts users* *’ continuous use intention for the app.*

Users fail to grasp clear rules for the use of personal information. Their perceived privacy uncertainty rises sharply. This situation becomes more obvious when service providers collect massive amounts of user data. It makes users attach greater importance to information control rights (Al-Natour et al., 2020). Improved perceived privacy control exists within online privacy environments. It means enterprises offer users more adjustable choices. Websites clearly notify users of cookie usage rules. Users will be more receptive to the collection and access of their personal information (Alkire et al., 2019). Subsequent research delivers further evidence. Information control-based privacy protection tools can effectively reduce users’ privacy concerns (Y. Li et al., 2020). Some platforms adopt highly transparent privacy policies. These policies bring two positive outcomes. They lift users’ perceived privacy control over personal data. They also mitigate negative psychological expectations arising from information collection. These effects jointly sustain users’ favorable attitudes toward app usage. Based on the above theoretical and empirical framework, the following hypothesis was developed:

**Hypothesis** **6.** *Information usage transparency positively influences users* *’ continuous use intention for the app through the mediating role of perceived privacy control.*

### 3.3. The Moderating Role of Text Information Sensitivity

Users’ reactions to privacy decisions are largely influenced by the type of information requested (Kitkowska et al., 2023). Risk attribute is an inborn feature of information. It has a positive correlation with individual information sensitivity. This attribute varies greatly across types of information. Most people regard financial data and personal identity information as high-risk information. Such information belongs to highly sensitive content. Purchasing records and lifestyle data carry lower sensitivity. Users are more willing to share this less sensitive information. Malhotra et al. put forward consistent findings. Users treat highly sensitive information with extra caution. They generate negative feelings about the use of such information. Some users even refuse relevant cooperation (Malhotra et al., 2004).

Some scholars research privacy policies. They claim that users’ attitudes toward privacy policies stem from their sensitivity to the information contained in these documents (Sheehan & Hoy, 2000). Communication privacy management theory provides relevant theoretical logic. The requested information carries low sensitivity. The type of information barely changes users’ views of privacy policies. Users are more willing to cooperate with service providers under this condition. Text information sensitivity is high. Users will focus more on the privacy policy text. They can clearly judge policy content yet hold low trust in service providers (Shezan et al., 2022).

Text information sensitivity can negatively moderate the correlation between information usage transparency and perceived privacy fatigue in online privacy environments. Two distinct scenarios explain this moderating effect. The first scenario involves highly sensitive information. Privacy policies with high transparency about information use rarely trigger perceived privacy fatigue among users. The second scenario involves low-sensitivity information. Highly transparent privacy policies significantly raise users’ perceived privacy fatigue. Based on the above theoretical and empirical framework, the following hypothesis was developed:

**Hypothesis** **7.** *Text information sensitivity negatively moderates the relationship between information usage transparency and perceived privacy fatigue. Specifically, compared with low text information sensitivity, the positive impact of high (vs. low) information usage transparency on perceived privacy fatigue is weaker under high text information sensitivity.*

In addition, text information sensitivity positively moderates the relationship between high information usage transparency and perceived privacy control. Studies show that highly sensitive information makes users value perceived privacy control more, so users’ perceived privacy control is stronger when text information is sensitive. Based on the above theoretical and empirical framework, the following hypothesis was developed:

**Hypothesis** **8.** *Text information sensitivity positively moderates the relationship between information usage transparency and perceived privacy control. Specifically, compared with low text information sensitivity, the positive impact of high (vs. low) information usage transparency on perceived privacy control is stronger under high text information sensitivity.*

**Figure 1:**
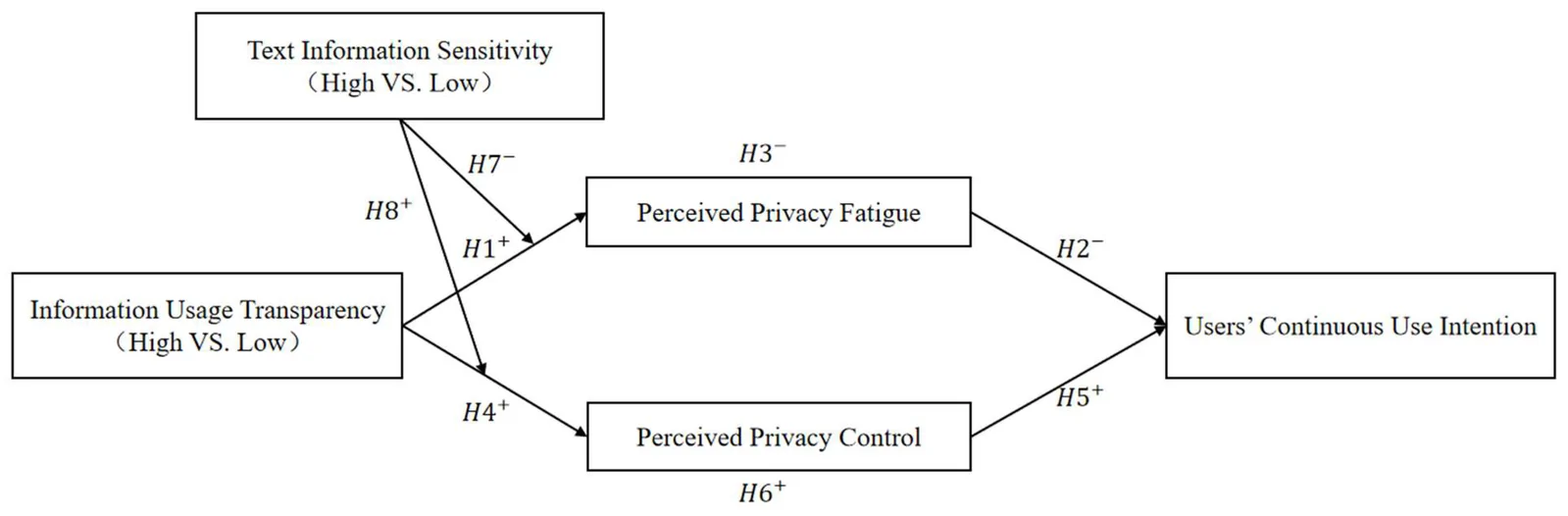
Research hypothesis model.

Based on the hypotheses above, the research model is shown in Figure 1.

## 4. Methodology

**Figure 2:**
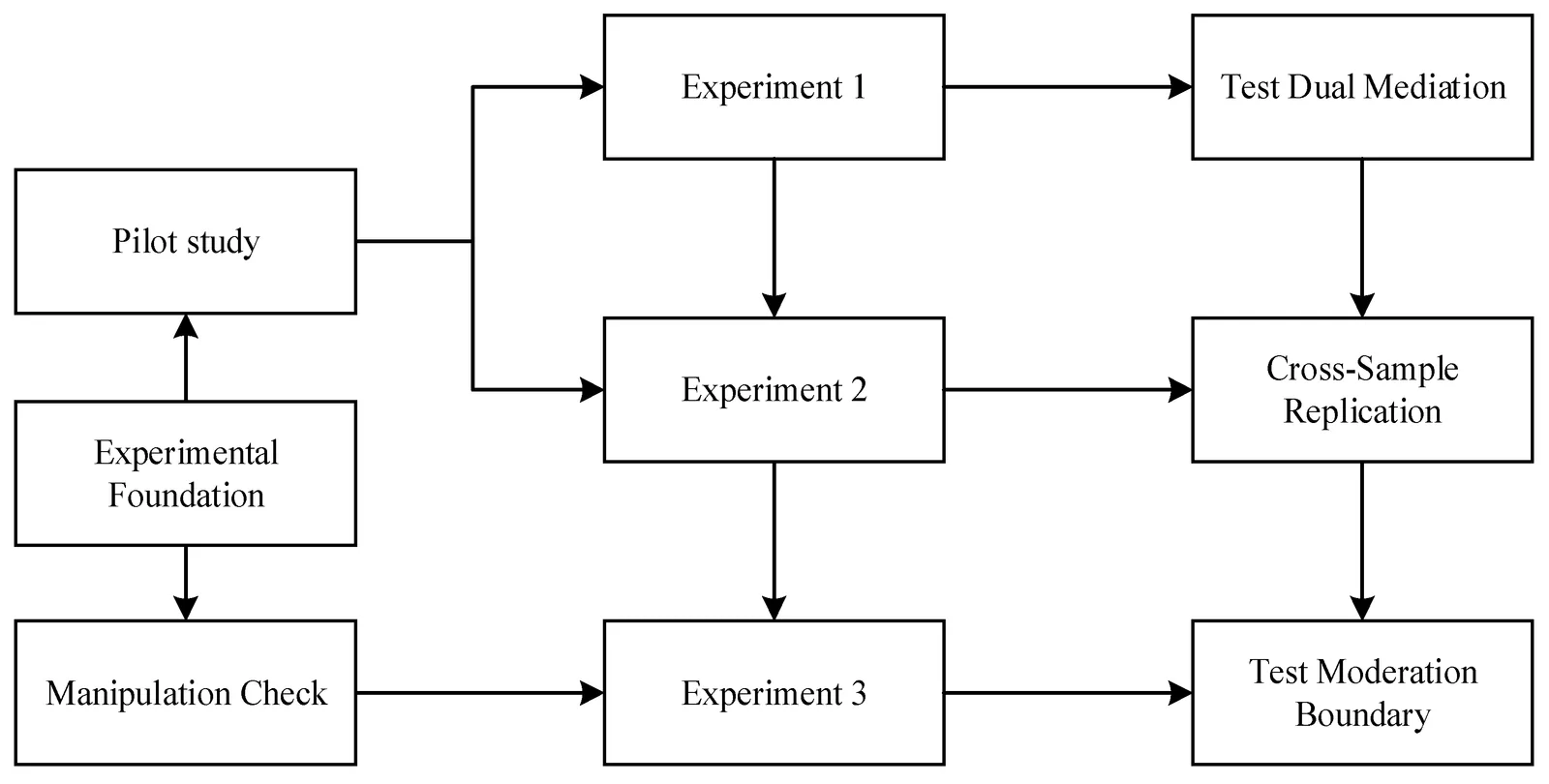
Schematic overview of experimental procedures.

Scenario experiments fit the research context. These experiments can manipulate situational variables. They simulate real decision-making scenarios. The controlled environment presents different external stimuli to participants. It records dynamic psychological and behavioral responses. These responses align with Stress and Coping Theory. This study includes one pilot study and three scenario-based experiments. The pilot study serves two purposes. It pretests experimental materials. It also verifies the manipulation of core variables. Experiment 1 examines the main effect of information-use transparency on continuance intention. It also tests two parallel mediators. The mediators are PPC and PPF. Experiment 2 uses a new independent sample to repeat the core effect. It further verifies the stability of the mediation mechanism. Experiment 3 applies a 2 × 2 factorial design. It explores the moderating effect of text information sensitivity. This step clarifies the boundary condition of the proposed relationship (see Figure 2).

### 4.1. Pilot Study

#### 4.1.1. Information Usage Transparency

This study selects the top 10 apps from the download charts of the App Store and the Huawei AppGallery. It conducts a detailed analysis of the privacy policies of these widely used apps. The analysis combines the frequency of permission requests. Six categories of information are finally chosen as the research focus. The categories include mobile phone numbers, storage space, location information, identity information, microphones, and contact lists.

Researchers adjust clauses within privacy policies. The adjusted content covers the types of information collected, the collection methods, the collection purposes, and the information disclosure rules. The adjustment targets the measurement of IUT. Two groups of privacy policy texts are formed after revision. One group features high IUT, and the other features low IUT.

A total of 39 college students (19 males and 20 females) participated in the evaluation task (see Table 1). Participants scored two versions of privacy policy texts via a 7-point Likert scale. The measurement item reads “I am very clear about how this mobile app uses my personal information” (Martin et al., 2017). The Cronbach’s alpha value of the scale reaches 0.95.

An independent-samples *t*-test produces clear statistical results. Participants in the high-transparency group obtain a significantly higher average Likert score (M_high = 4.95) than participants in the low-transparency group (M_low = 2.62). The test results show (t = −5.57, *p* < 0.01). Large gaps exist between the scores of high-transparency and low-transparency privacy policy materials. The outcome demonstrates successful manipulation of information-use transparency in the experiment. Supplementary nonparametric tests are conducted to assess the stability of the experimental results. Table 2 records matching consistent outcomes.

#### 4.1.2. Text Information Sensitivity

For the six types of information selected above—mobile phone numbers, storage space, location data, identity information, microphones, and contact lists—which are frequently requested in combination, this study drew upon the findings of Yang and Wang (2009) to distinguish between the varying levels of sensitivity of these data points. A group of 10 college students was recruited to score the sensitivity of the above information with a 7-point Likert scale. Researchers analyzed the scoring results and open-ended feedback submitted by these participants. Ambiguous expressions in the scenario materials have been revised. Item wording was adjusted to reduce inconsistent subjective scoring before the formal manipulation check. This small preliminary sample brings two practical benefits. It polishes experimental stimuli. It also removes obvious flaws in experimental materials with low time and resource costs. Revisions are finished after the preliminary evaluation. Mobile phone numbers, identity information, and contact lists are categorized as high-sensitivity information. Storage space, location data, and microphone access fall into the category of low-sensitivity information. A new independent sample of 30 participants is recruited for the formal manipulation check. No participants overlap with the 10 raters in the preliminary stage. All evaluation work relies on the revised experimental materials. This two-stage evaluation process ensures the reliability and validity of manipulations adopted in subsequent main experiments. The formal evaluation sample includes 30 college students (16 males and 14 females). None of these participants took part in the preliminary rater group (see Table 3). These participants completed the TIS Scale (7-point scale) to evaluate the sensitivity of the two information groups. The scale item reads: “From the perspective of personal privacy, this type of information is highly sensitive in an online environment” (Castañeda & Montoro, 2007). The Cronbach’s alpha value of the scale equals 0.91.

An independent samples *t*-test is conducted to verify the manipulation effect. Participants in the high-transparency group obtain a significantly higher average Likert score (M_high = 5.24) than participants in the low-transparency group (M_low = 2.56). The test results show (t = −6.70, *p* < 0.01). This statistical outcome demonstrates the effective manipulation of information-use transparency. Supplementary nonparametric tests are conducted to further assess the stability of the manipulation effect. Consistent test results are presented in Table 4.

### 4.2. Experiment 1

#### 4.2.1. Experimental Design

Experiment 1 adopts a single-factor between-subjects design. It tests the double-edged effect of information-use transparency on CUI. The two experimental conditions are high transparency and low transparency. Standardized experimental stimulus materials simulate real app usage scenarios for data collection. Such materials help improve external validity (Breves et al., 2019). Multiple strict control measures are implemented throughout the entire experimental process. These measures ensure good internal validity. The specific control arrangements are listed below. All participants are randomly assigned to different experimental groups. All participants receive identical experimental instructions. The questionnaire structure stays consistent for every participant. Neutral invented app names are used. These names carry no real brand influences. The setting removes bias caused by brand preferences. Invalid questionnaires and participants who participated in the pilot study are excluded. Formal manipulation checks are set to confirm successful manipulation of core variables. Gender, age, and educational background are set as control variables in the regression analysis. The step excludes interference from confounding factors. The recruitment pool contained 165 participants in total. A total of 147 valid samples were retained after filtering invalid responses (see Table 5). All participants from the pilot study were excluded from this sample.

#### 4.2.2. Stimulus Materials

**Figure 3:**
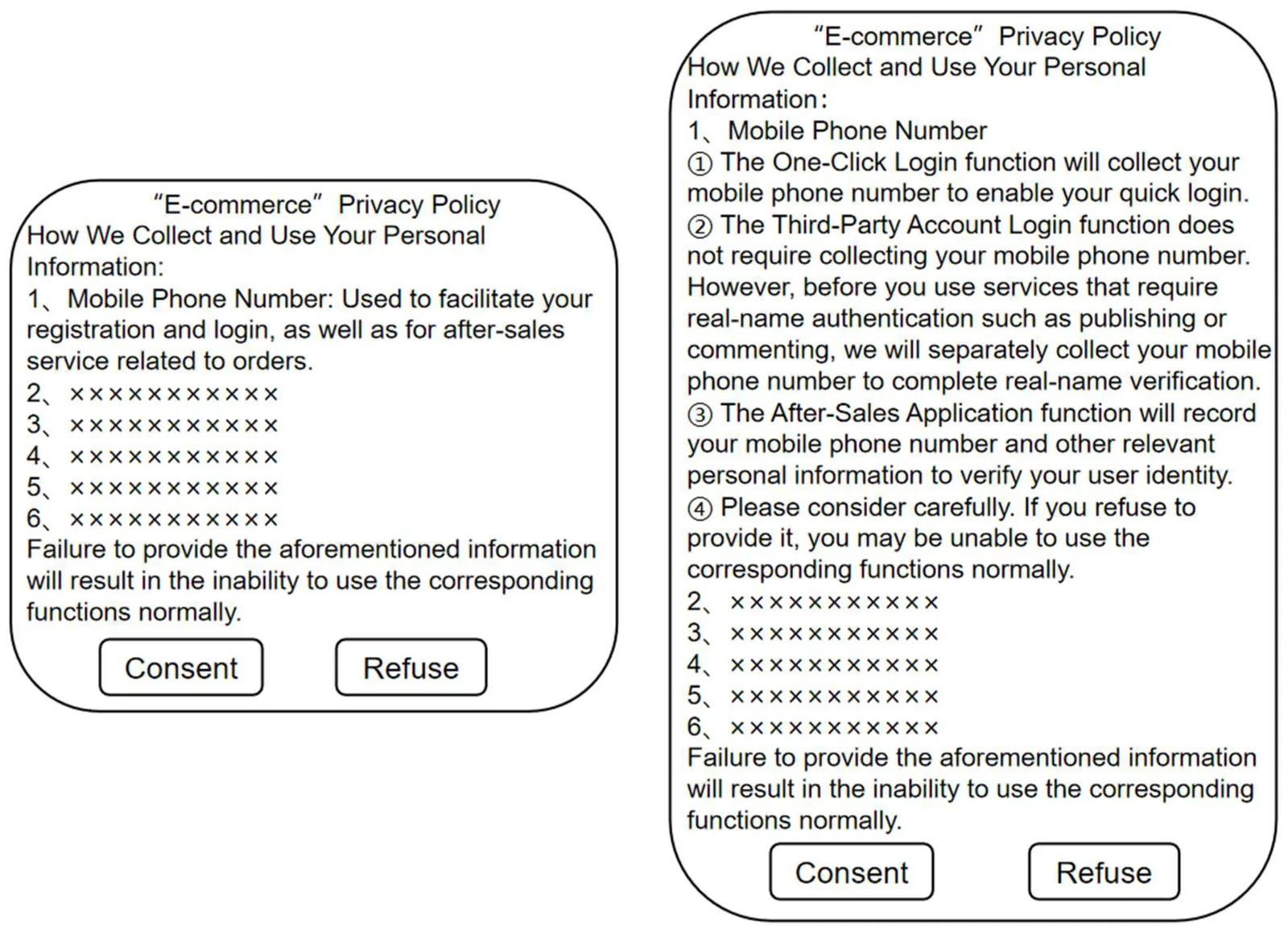
Difference in information-use transparency (low on left, high on right).

This study picks the authorization page of an e-commerce app as stimulus material. The virtual e-commerce app is named “E-commerce”. This naming rule avoids the impact of brand recognition and brand trust from real e-commerce platforms on experimental outcomes. Participants are randomly allocated into two experimental groups. All participants receive unified instructions before the experiment starts. The instruction content reads: “Suppose you have just finished downloading and are about to use an e-commerce app called ‘E-commerce’.” Two versions of privacy policy texts are provided to all participants. The two versions differ in the transparency of information use. Participants need to read the provided texts. All participants complete the reading task. They then tick the checkbox marked “I have read”. This operation grants them formal access to the “E-commerce” app (see Figure 3).

#### 4.2.3. Measurements

Participants finish reading the stimulus materials. They then fill in questionnaire items. These items measure PPF, PPC, users’ continuance intention toward apps, and demographic information. All questionnaire items are derived from validated and established scales: PPC (Mothersbaugh et al., 2012), PPF (Chaouali, 2016), and CUI (Bhattacherjee, 2001) (see Table 6). Detailed scale items are listed in Appendix A Table A1 (The same below). Gender, age, and education are common control variables in scenario-based experimental research (Breves et al., 2019). Demographic features can influence users’ privacy perceptions and usage tendencies. This study uses these identical control variables. The setting removes confounding bias in estimating causal paths.

Table 7 lists Fornell–Larcker discriminant validity outcomes. Diagonal bold values stand for the square root of each construct’s AVE. Off-diagonal entries record correlations between latent composite scores. Each AVE square root exceeds all cross-construct correlation coefficients. Three latent constructs meet discriminant validity criteria. All latent correlations reach the 0.01 significance level.

Table 8 lists descriptive statistics and full-variable Pearson correlations. Mean and standard deviation reflect the sample distribution of all indicators. IUT correlates positively with PPC and PPF. The direct correlation between IUT and CUI lacks significance. Two opposite indirect effects neutralize the total crude correlation.

#### 4.2.4. Results

Before the between-group comparison, Kolmogorov–Smirnov normality and Levene homogeneity tests were conducted for PPC and PPF. Full test statistics are summarized in Appendix A Table A2. Minor portions of the measured variables failed the strict normality test (*p* < 0.05), yet all measurement items complied with Kline’s approximate normality criterion that absolute skewness and excess kurtosis values stay below 3 and 10, respectively (skewness: 0.21–0.60; excess kurtosis: 0.05–0.73) (Kline, 2023). Combined with the large sample (*N* = 147), the Central Limit Theorem supports parametric one-way ANOVA as a robustness benchmark (W. Zhang et al., 2025). Levene’s outputs were PPC (F(1, 145) = 0.77, *p* = 0.38) and PPF (F(1, 145) = 3.23, *p* = 0.08), confirming equal variances across groups. Distribution-free Mann–Whitney U served as the primary analytical framework to avoid normality bias, with one-way ANOVA added for cross-verification of group difference patterns. The parametric ANOVA yielded consistent group distinctions: participants under high transparency presented significantly higher PPC (M_high = 4.56, SD_high = 1.32 vs. M_low = 3.97, SD_low = 1.14; F(1, 145) = 8.22, *p* < 0.01) and stronger PPF (M_high = 4.69, SD_high = 1.37 vs. M_low = 4.14, SD_low = 0.01). Significant intergroup disparities from Mann–Whitney U tests aligned with the above ANOVA outcomes: PPC (Z = −3.28, *p* < 0.01) and PPF (Z = −2.40, *p* < 0.05) (see Table 9). The consistent results from two analytical methods jointly validated Hypotheses 1 and 4.

Next, four multiple linear regression models (M1–M4) were constructed with CUI as the dependent variable. M1 is the baseline model with only control variables (gender, age, and education level). M2 adds PPF as a predictor of continuance intention. M3 adds PPC as a predictor of continuance intention. M4 is the full model in which both mediators (PPF and PPC) simultaneously predict continuance intention. All models were estimated using Ordinary Least Squares (OLS). Collinearity diagnostics indicated that all Variance Inflation Factor (VIF) values were below 5, suggesting no severe multicollinearity among predictors. Table 10 reports the estimation results for all four models. The baseline model (M1) shows negligible explanatory power (adjusted R^2^ = −0.006, F = 0.73, *p* > 0.05). When entered separately, PPF (M2: adjusted R^2^ = 0.19) and PPC (M3: adjusted R^2^ = 0.41) each significantly predict continuance intention, with PPF exhibiting a negative effect (β = −0.41, *p* < 0.001) and PPC exhibiting a positive effect (β = 0.61, *p* < 0.001), thereby supporting Hypotheses 2 and 5. In the full model (M4), which includes both mediators simultaneously, the overall explanatory power increases further (adjusted R^2^ = 0.47, F = 27.19, *p* < 0.001). Notably, in M4, the coefficient of PPF becomes positive (β = 0.54, *p* < 0.001), while that of PPC becomes negative (β = −0.25, *p* < 0.001), reflecting a mutual suppression effect between the two mediators after controlling for their shared variance. All demographic control variables remain insignificant across all models, ruling out confounding interference from demographic factors.

The PROCESS macro is adopted to test the mediating effects of PPC and PPF (Hayes, 2017). Gender, age, and education level are set as demographic control variables in the analysis. The analytical outcomes are recorded in Table 11 and present two key mediating results. First, PPF carries a significant mediating role. It transmits the negative influence of IUT on users’ continuance intention. The indirect effect value equals −0.13. The 95% bias-corrected confidence interval is [−0.28, −0.02]. The confidence interval excludes zero. Hypothesis 3 receives support from this result. Second, PPC generates a significant mediating effect. It mediates the positive influence of IUT on users’ continuance intention. The indirect effect size reaches 0.31. The corresponding 95% confidence interval is [0.08, 0.57]. This interval does not contain zero, which verifies Hypothesis 6. An additional comparison of the two indirect effects yields further findings. The absolute value of the indirect effect through PPC is significantly greater than that through PPF. The 95% confidence interval for the gap between the two indirect effects is [0.19, 0.68]. This interval excludes zero. The two mediating paths function in opposite directions. PPC positively boosts users’ intention to continue use. PPF negatively affects users’ intention to continue use. The significant statistical gap only illustrates one point. The positive indirect path produces a stronger absolute net influence on continuance intention.

### 4.3. Experiment 2

#### 4.3.1. Experimental Design

Two forms of authorization exist in practical scenarios. The two categories are general authorization and scenario-based authorization. The two authorization methods may create different impacts on users’ information disclosure behaviors and their evaluations of mobile applications. Experiment 2 verifies the robustness of the double-edged sword effect. The experiment uses the privacy policies of instant messaging apps as stimuli. It replicates the results of Experiment 1. It also checks whether identical conclusions can be obtained under different app contexts. Experiment 2 follows the same setup as Experiment 1. Standardized simulated app scenario materials are used to guarantee external validity. A comprehensive set of strict, unified control measures is implemented throughout the experiment. These measures sustain high internal validity. Experiment 2 adopts a single-factor between-subjects design. The two conditions are high and low IUT. A communication app serves as the research context for replicating Experiment 1 and testing robustness. A total of 203 participants were recruited for Experiment 2. A total of 182 valid samples were retained after screening invalid responses (see Table 12). All participants who joined prior experimental stages were excluded from this sample.

#### 4.3.2. Stimulus Materials

**Figure 4:**
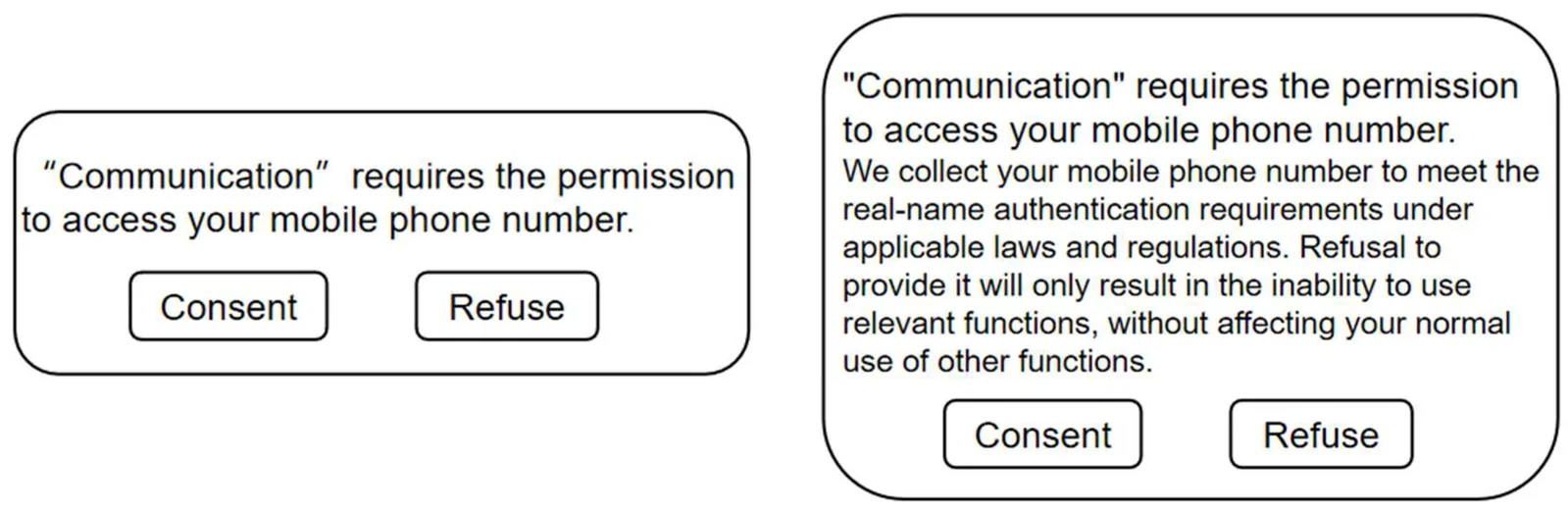
Scenario authorization difference in information-use transparency (low on left, high on right).

Experiment 2 sets a fictional app named “Communication” as the research stimulus (see Figure 4). Participants are randomly divided into two experimental groups under the scenario-based authorization condition. All participants read unified instructions at the beginning of the experiment. The instruction text reads: “You may now start using Communication.” Participants receive subsequent notifications. Pop-up windows will emerge when users open specific app functions. These windows display privacy policies with varying levels of IUT. Participants need to read the presented content. All participants complete the reading task. They then tick the checkbox marked “I have read”.

#### 4.3.3. Measurements

Scales were identical to Experiment 1: PPF (Chaouali, 2016); PPC (Mothersbaugh et al., 2012); and CUI (Bhattacherjee, 2001) (see Table 13). Gender, age, and educational attainment were included as control variables (Breves et al., 2019).

Table 14 reports Fornell–Larcker discriminant validity results. Bold diagonal values stand for the square root of each construct’s AVE. Off-diagonal entries show latent variable correlations. Each AVE square root exceeds cross-construct correlation coefficients. All three constructs reach acceptable discriminant validity. All latent correlations reach the 0.01 significance level.

Table 15 lists descriptive statistics and full-variable Pearson correlations. Mean and standard deviation reflect the sample distribution of all indicators. IUT correlates positively with PPC and PPF. The direct correlation between IUT and CUI lacks significance. Two opposite indirect effects neutralize the total crude correlation.

#### 4.3.4. Results

Prior to between-group comparison, Kolmogorov–Smirnov normality and Levene homogeneity tests were conducted for PPC and PPF. Full test statistics are summarized in Appendix A Table A3. Minor portions of the measured variables failed the strict normality test (*p* < 0.05), yet all measurement items complied with Kline’s approximate normality criterion that absolute skewness and excess kurtosis values stay below 3 and 10, respectively (skewness: 0.06–0.67; excess kurtosis: 0.05–0.79) (Kline, 2023). Combined with the large sample (*N* = 182), the Central Limit Theorem supports parametric one-way ANOVA as a robustness benchmark (W. Zhang et al., 2025). Levene’s outputs were PPC (F(1, 180) = 1.72, *p* = 0.19) and PPF (F(1, 180) = 3.64, *p* = 0.06), confirming equal variances across groups. Distribution-free Mann–Whitney U served as the primary analytical framework to avoid normality bias, with one-way ANOVA added for cross-verification of group difference patterns. The parametric ANOVA yielded consistent group distinctions: participants under high transparency presented significantly higher PPC (M_high = 4.42, SD_high = 1.21 vs. M_low = 3.82, SD_low = 1.01; F(1, 180) = 12.64, *p* < 0.01) and stronger PPF (M_high = 4.54, SD_high = 1.25 vs. M_low = 4.01, SD_low = 1.11; F(1, 180) = 9.03, *p* < 0.01). Significant intergroup disparities from Mann–Whitney U aligned with the above ANOVA outcomes: PPC (Z = −3.92, *p* < 0.001) and PPF (Z = −2.75, *p* < 0.01) (see Table 16). The consistent results from two analytical methods further validated Hypotheses 1 and 4.

Ordinary Least Squares (OLS) multiple linear regression was used to estimate the regression coefficients. Four multiple linear regression models (M1–M4) were constructed with CUI as the dependent variable. M1 is the baseline model with only control variables (gender, age, and education level). M2 adds PPF as a predictor of continuance intention. M3 adds PPC as a predictor of continuance intention. M4 is the full model in which both mediators (PPF and PPC) simultaneously predict continuance intention. Collinearity diagnostics indicated that all Variance Inflation Factor (VIF) values were below 5, suggesting no severe multicollinearity among predictors. Table 17 reports the estimation results for all four models. The baseline model (M1) shows negligible explanatory power (adjusted R^2^ = −0.009, F = 0.49, *p* > 0.05). When entered separately, PPF (M2: adjusted R^2^ = 0.10) and PPC (M3: adjusted R^2^ = 0.22) each significantly predict continuance intention, with PPF exhibiting a negative effect (β = −0.32, *p* < 0.01) and PPC exhibiting a positive effect (β = 0.49, *p* < 0.01), thereby supporting Hypotheses 2 and 5. In the full model (M4), which includes both mediators simultaneously, the overall explanatory power increases further (adjusted R^2^ = 0.26, F = 13.76, *p* < 0.001). In M4, PPF remains a significant negative predictor (β = −0.22, *p* < 0.001) and PPC remains a significant positive predictor (β = 0.43, *p* < 0.001), though the magnitude of both coefficients is reduced compared to their respective separate models, reflecting the mutual suppression effect between the two mediators after controlling for their shared variance. All demographic control variables remain insignificant across all models, ruling out confounding interference from demographic factors. This conclusion matches the experimental outcome of Experiment 1.

The PROCESS macro is adopted to test the mediating effects of PPC and PPF (Hayes, 2017). Gender, age, and education level are treated as demographic control variables in the analysis. Table 18 records the analytical results. The negative path runs from IUT to users’ continuance intention. Its indirect effect value equals −0.09. The corresponding 95% bias-corrected confidence interval is [−0.21, −0.01]. This confidence interval excludes zero. The outcome further verifies Hypothesis 3. The positive path runs from IUT to users’ continuance intention. Its indirect effect size reaches 0.25. The corresponding 95% confidence interval is [0.11, 0.42]. This interval does not contain zero, which further validates Hypothesis 6. A comparison of the two mediating effects produces consistent findings. The absolute value of the indirect effect mediated by PPC is significantly greater than that mediated by PPF. The 95% confidence interval for the difference between the two indirect effects is [0.19, 0.50]. This interval excludes zero. The two mediating paths work in opposite directions. PPC positively improves users’ CUI. PPF negatively restrains users’ intention to continue use. The significant statistical gap merely illustrates one point. The positive indirect path creates a stronger absolute net influence on continuance intention.

### 4.4. Experiment 3

#### 4.4.1. Experimental Design

Experiment 3 examined the moderating role of TIS using a 2 (IUT: high vs. low) × 2 (TIS: high vs. low) between-subjects design. A total of 223 participants were recruited; 200 valid samples remained (see Table 19). Participants from previous studies were excluded.

#### 4.4.2. Stimulus Materials

Experiment 3 adopts the same stimulus materials as Experiment 1. The authorization page of the e-commerce app “E-commerce” serves as the stimulus material. Participants are randomly allocated into four experimental groups. All participants read unified instructions before the formal experiment begins. The instruction reads: “Suppose you have just finished downloading and are about to use the e-commerce app named ‘E-commerce’”.

All participants receive privacy policy texts after the instruction session. These texts differ in the transparency of information use and the sensitivity of textual information. Participants need to read the provided texts carefully. Two versions of privacy policy materials are prepared based on two levels of TIS. The combination generates four distinct questionnaire versions. These questionnaires are randomly distributed to participants. Each participant fills out the assigned questionnaire.

#### 4.4.3. Measurements

Scales were identical to Experiment 1: PPF (Chaouali, 2016); PPC (Mothersbaugh et al., 2012); and CUI (Bhattacherjee, 2001) (see Table 20). Gender, age, and educational attainment were included as control variables (Breves et al., 2019).

Table 21 presents Fornell–Larcker discriminant validity results. Bold numbers equal the square root of each construct’s AVE. Off-diagonal cells record latent correlation coefficients. Each AVE square root exceeds cross-construct correlations. Three latent constructs meet discriminant validity criteria. All latent correlations reach the 0.01 significance level.

Table 22 lists descriptive statistics and full-variable Pearson correlations. Mean and standard deviation reflect the sample distribution of all indicators. IUT correlates positively with PPC and PPF. TIS correlates positively with PPC and negatively with PPF. IUT holds a weakly significant positive link with CUI. TIS shows no significant correlation with CUI.

#### 4.4.4. Results

① Manipulation Check

Manipulation checks for IUT and TIS are implemented before the main experiment. A total of 44 participants took part in the evaluation task of Experiment 3. Four groups of privacy policy texts are formed through two dimensions. The dimensions include IUT (high vs. low) and TIS (high vs. low). Participants score the four privacy policy texts on a 7-point Likert scale (see Table 23). The measurement item for IUT originates from Martin et al. (2017), with Cronbach’s alpha = 0.93. The measurement item for TIS originates from Castañeda and Montoro (2007), with Cronbach’s alpha = 0.92.

Results from an independent samples *t*-test showed that high text sensitivity (M_high = 5.15) was significantly higher than low text sensitivity (M_low = 2.77), with t = −7.79 and *p* < 0.01. Furthermore, low information-use transparency (M_low = 2.53) was significantly lower than high information-use transparency (M_high = 5.19), with t = −8.36 and *p* < 0.01. Therefore, the manipulation of information-use transparency and text sensitivity in this experiment was successful. To verify the robustness of our findings, supplementary nonparametric tests were conducted, and consistent results are presented in Table 24.

② Hypothesis Testing

Before the between-group comparison for Experiment 3, Kolmogorov–Smirnov normality and Levene homogeneity tests were implemented on PPC and PPF, with all test results compiled in Appendix A Table A4. Minor parts of the measured variables failed strict K-S normality tests (*p* < 0.05), yet all composite construct scores satisfied Kline’s (2023) approximate normality thresholds: absolute skewness between 0.06 and 0.30, and absolute excess kurtosis ranging from 0.88 to 10. Combined with the large sample size (*N* = 200), the Central Limit Theorem justifies parametric testing as a robustness reference (W. Zhang et al., 2025). Levene’s test results were PPC (F(1, 198) = 20.31, *p* < 0.05) and PPF (F(1, 198) = 1.03, *p* = 0.31), indicating heterogeneous variances for PPC and homogeneous variances for PPF. A distribution-free rank-transformed Scheirer–Ray–Hare two-way nonparametric test was adopted as the primary analytical tool to eliminate normality and variance heterogeneity interference. In contrast, standard two-way ANOVA for PPC and PPF served as supplementary cross-verification. Separate one-way ANOVAs were first conducted to replicate the baseline effects observed in Experiment 1 and Experiment 3. The parametric tests revealed consistent group differences: high transparency generated significantly stronger PPC (M_high = 4.75, SD_high = 1.53 vs. M_low = 4.09, SD_low = 1.09; F(1, 198) = 12.32, *p* < 0.001) and higher PPF (M_high = 4.60, SD_high = 1.13 vs. M_low = 4.14, SD_low = 1.18; F(1, 198) = 7.76, *p* < 0.001). Significant intergroup gaps detected by nonparametric analysis matched the ANOVA outputs (PPC Z = −3.33, *p* < 0.01; PPF Z = −2.44, *p* < 0.05, see Table 25). Consistent findings across parametric and nonparametric analyses further validate Hypotheses 1 and 4.

Ordinary Least Squares (OLS) multiple linear regression was used to test the theoretical model. Four multiple linear regression models (M1–M4) were constructed with CUI as the dependent variable. M1 is the baseline model with only control variables (gender, age, and education level). M2 adds PPF as a predictor of continuance intention. M3 adds PPC as a predictor of continuance intention. M4 is the full model in which both mediators (PPF and PPC) simultaneously predict continuance intention. Collinearity diagnostics indicated that all Variance Inflation Factor (VIF) values were below 5, suggesting no severe multicollinearity among predictors. Table 26 reports the estimation results for all four models. The baseline model (M1) shows negligible explanatory power (adjusted R^2^ = −0.008, F = 0.48, *p* > 0.05). When entered separately, PPF (M2: adjusted R^2^ = 0.11) and PPC (M3: adjusted R^2^ = 0.16) each significantly predict continuance intention, with PPF exhibiting a negative effect (β = −0.45, *p* < 0.001) and PPC exhibiting a positive effect (β = 0.46, *p* < 0.001), thereby supporting Hypotheses 2 and 5. In the full model (M4), which includes both mediators simultaneously, the overall explanatory power increases substantially (adjusted R^2^ = 0.35, F = 22.41, *p* < 0.001). Notably, in M4, the negative effect of PPF is amplified (β = −0.59, *p* < 0.001) and the positive effect of PPC is also strengthened (β = 0.56, *p* < 0.001) compared to their respective separate models, reflecting the mutual enhancement effect between the two mediators after controlling for their shared variance. All demographic control variables remain insignificant across all models, ruling out confounding interference from demographic factors. This statistical outcome is consistent with the findings of Experiment 1.

The PROCESS macro (Model 4) from Hayes (2017) is adopted for further analysis. The analysis strictly tests the mediating roles of PPC and PPF. Gender, age, and educational attainment are defined as demographic variables to eliminate potential confounding. Table 27 records the analytical results. IUT affects users’ continuance intention through two significant mediating paths. The first path is the negative suppression path. PPF delivers a mediating effect of −0.28. Its 95% bias-corrected Bootstrap confidence interval equals [−0.51, −0.09]. The interval excludes zero. Hypothesis 3 receives empirical support accordingly. The second path is the positive promotion path. PPC produces a mediating effect size of 0.35. Its 95% confidence interval equals [0.15, 0.56]. This interval does not contain zero, which verifies Hypothesis 6. An additional comparison is conducted on the two indirect effect values. The 95% confidence interval for the difference between indirect effects is [0.31, 0.97]. The absolute value of the indirect effect mediated by PPC is significantly greater than that mediated by PPF. The two mediating paths work in opposite directions. PPC positively boosts users’ intention to continue use. PPF negatively affects users’ intention to continue use. The significant gap between the two indirect effects only illustrates one point. The positive mediating path brings a stronger absolute net influence on continuance intention.

PPF served as the dependent variable. A rank-transformed Scheirer–Ray–Hare nonparametric test was adopted to handle non-normal and heterogeneous variance data, with a 2 (IUT: high vs. low) × 2 (TIS: high vs. low) factorial design to test the moderating effect of TIS. Significant main effects of IUT (H = 1.47, *p* < 0.05) and TIS (H = 1.19, *p* < 0.05) were observed. The IUT × TIS interaction was statistically significant (H = 1.11, *p* < 0.05). Further Kruskal–Wallis testing revealed overall differences across the four experimental groups (H = 15.21, *p* < 0.01). Dunn’s adjusted pairwise comparisons clarified group differences. The high-IUT and low-TIS group exhibited significantly higher PPF. This group differed from the low-IUT and low-TIS group (*p* < 0.01) and the low-IUT and high-TIS group (*p* < 0.01). It also differed significantly from the high-IUT and high-TIS group (*p* < 0.05). No meaningful disparities existed among the remaining three groups (all *p* > 0.05). Simple effect analyses based on raw scores were further conducted to unpack the significant interaction. Within the high-TIS group, IUT exerted no significant influence on PPF. High IUT yields M_high = 4.25 and SD_high = 0.16. Low IUT yields M_low = 4.15 and SD_low = 0.16. The test result F(1, 198) = 0.17 and *p* = 0.68. The difference lacks statistical significance. The low-TIS group presents a significant main effect of IUT (F(1, 198) = 12.96, *p* < 0.01). For privacy policy texts containing low-sensitivity information, the gap in PPF between high and low IUT is obvious. The high-transparency average score M_high = 4.95, SD_high = 0.16. The low-transparency average score is M_low = 4.13, SD_low = 0.16.

**Figure 5:**
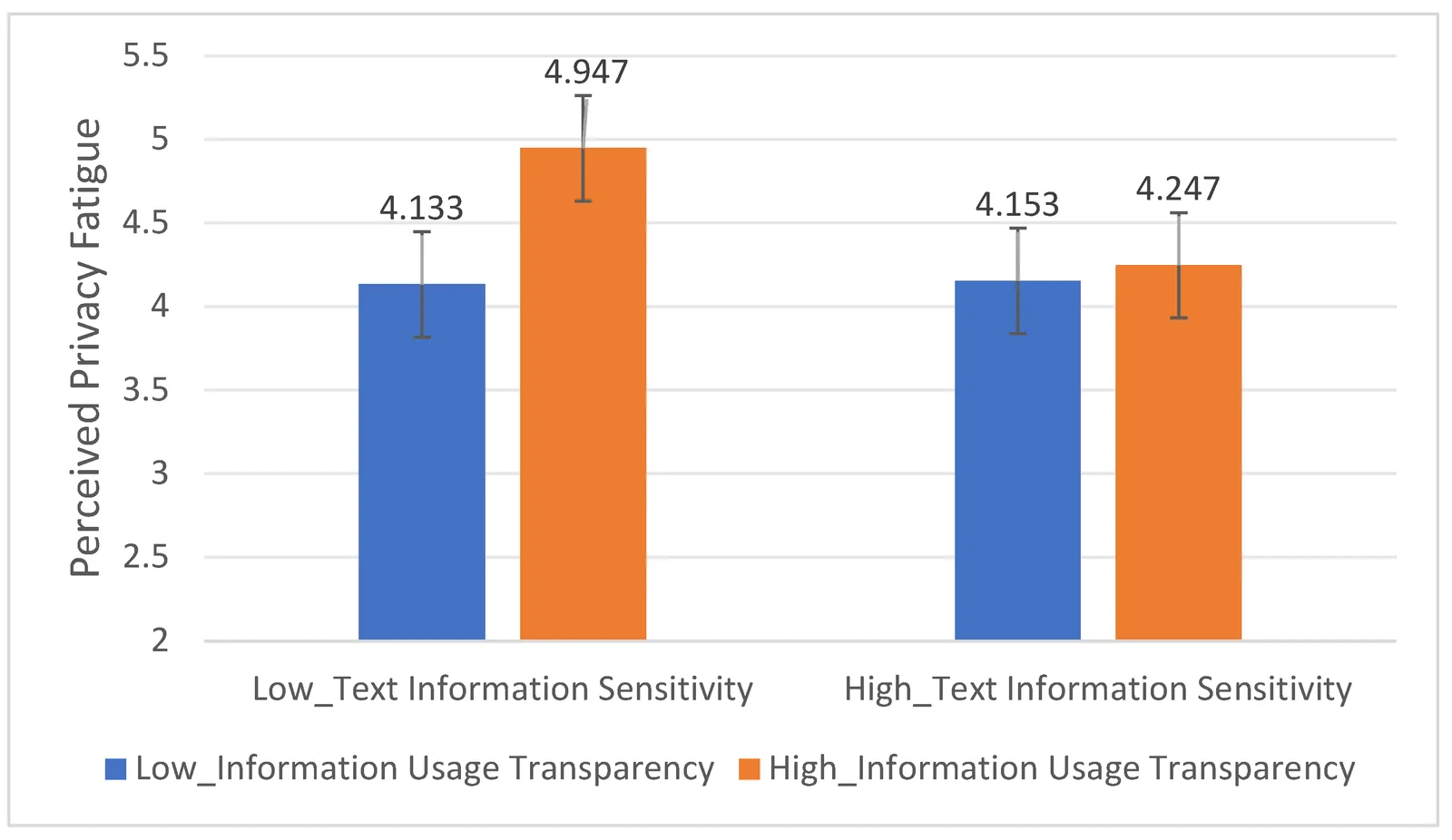
The moderating effect of text information sensitivity on the relationship between information-use transparency and perceived privacy fatigue.

Hayes’ PROCESS Model 7 runs conditional simple slope tests. The test outcomes match the ANOVA results and quantify the inflection point of this interaction. The simple slope of IUT predicting PPF is strongly positive and significant under low TIS (b = 0.79, SE = 0.23, t = 3.48, *p* < 0.001). The simple slope becomes small and non-significant under high TIS (b = 0.1164, SE = 0.23, t = 0.51, *p* = 0.61). The distinct contrast between the two slopes clarifies a clear boundary condition for the negative path of the double-edged sword effect. High IUT can amplify PPF. This amplifying effect largely fades and loses statistical significance when TIS is high. The moderating influence of TIS follows a clear pattern. Low-sensitivity information produces a stronger moderating effect on the link between IUT and PPF. High-sensitivity information generates a weaker moderating effect on this relationship. Figure 5 visualizes these empirical outcomes. Hypothesis 7 receives full empirical support.

PPC served as the dependent variable. A rank-transformed Scheirer–Ray–Hare nonparametric test was adopted to handle non-normal and heterogeneous variance data, with a 2 (IUT: high vs. low) × 2 (TIS: high vs. low) factorial design to test the positive moderating effect of TIS. Significant main effects of IUT (H = 2.75, *p* < 0.001) and TIS (H = 1.06, *p* < 0.05) were observed. The IUT × TIS interaction was statistically significant (H = 1.20, *p* < 0.05). Further Kruskal–Wallis testing revealed overall differences across the four experimental groups (H = 20.16, *p* < 0.001). Dunn’s adjusted pairwise comparisons clarified group differences. Only the high-IUT and high-TIS group exhibited significantly higher PPC. This group differed from the low-IUT and low-TIS group (*p* < 0.001), the high-IUT and low-TIS group (*p* < 0.05), and the low-IUT and high-TIS group (*p* < 0.001). No meaningful disparities existed among the remaining three groups (all *p* > 0.05). Simple effect analyses based on raw scores were further conducted to unpack the significant interaction. The high-IUT group has M_high = 4.34 and SD_high = 0.18. The low-IUT group has M_low = 4.10 and SD_low = 0.18. F(1, 198) = 0.82, *p* = 0.37. In the high-TIS group, high IUT yields significantly stronger PPC than low IUT: M_high = 5.17, SD_high = 0.18; M_low = 4.09, SD_low = 0.18; and F(1, 198) = 17.31, *p* < 0.01.

**Figure 6:**
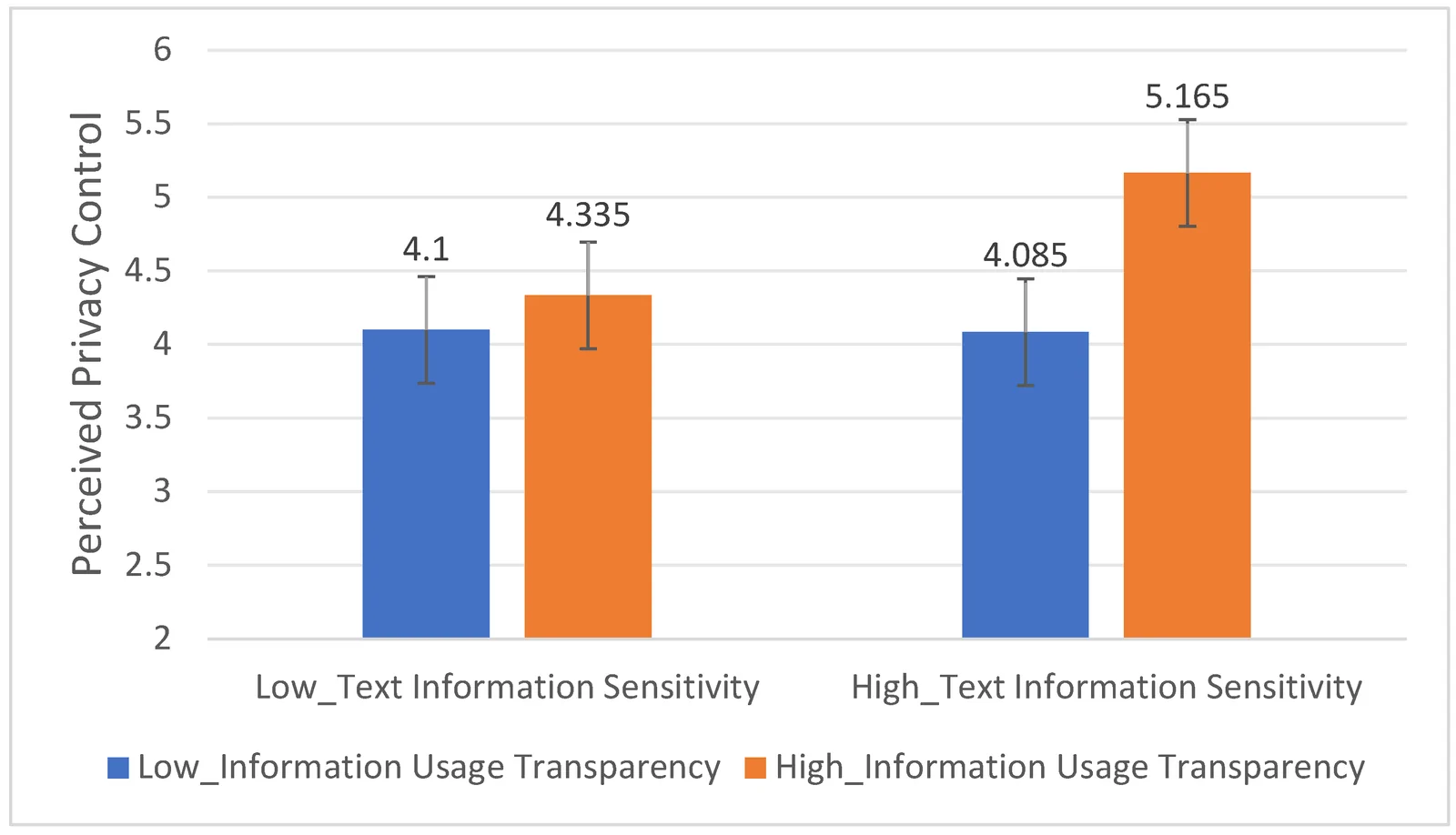
The moderating effect of text information sensitivity on the relationship between information-use transparency and perceived privacy control.

The PROCESS Model 7 is adopted to conduct a conditional simple slope analysis. The test further verifies the interaction pattern and identifies the inflection point of the positive boosting effect of IUT. The simple slope between IUT and PPC is weak and non-significant under low TIS (b = 0.23, SE = 0.26, t = 0.89, *p* = 0.38). The slope becomes strong and statistically significant under high TIS (b = 1.08, SE = 0.26, t = 4.11, *p* < 0.001). This obvious contrast clarifies a critical boundary condition of the double-edged sword mechanism. IUT can substantially increase PPC only when the sensitivity of text information is high. Figure 6 presents the full, detailed analytical results. Hypothesis 8 receives empirical support from these outcomes.

The moderated mediating effect receives further verification. The analytical method follows suggestions proposed by Preacher et al. (2007). The PROCESS program is adopted to test the research model. A total of 5000 repeated-sample simulations are conducted, and 95% confidence intervals are estimated. Table 28 reports the test results for the indirect path mediated by PPF. The indirect effect value under low TIS equals −0.49. Its 95% confidence interval is [−0.86, −0.17]. The interval excludes zero. The indirect effect reaches statistical significance. The indirect effect value under high TIS equals −0.07. Its 95% confidence interval is [−0.32, 0.18]. The interval contains zero. The indirect effect lacks statistical significance. The value of the moderated mediation effect equals 0.42. Its confidence interval is [0.019, 0.86]. The interval excludes zero. The moderated mediation effect is statistically significant.

Another set of results describes the positive indirect path transmitted through PPC. The indirect effect under low TIS equals 0.12. Its confidence interval is [−0.16, 0.40]. The interval contains zero. This indirect effect shows no significance. The indirect effect under high TIS equals 0.57. Its confidence interval is [0.29, 0.89]. The interval excludes zero. This indirect effect is significant. The moderated mediation effect value reaches 0.45. Its confidence interval is [0.05, 0.89]. The interval excludes zero. The moderated mediation effect achieves significance.

Two distinct patterns appear at different levels of TIS. High TIS strengthens the positive mediating role of PPC in the link between IUT and users’ continuance intention. High TIS weakens the negative mediating role of PPF in this relationship. TIS acts as a critical boundary condition. It controls the occurrence of the double-edged sword effect brought by IUT.

## 5. Conclusions and Discussion

### 5.1. Research Conclusions

All empirical studies draw several core conclusions. The conclusions center on the double-edged sword effect of information-use transparency in privacy policies. This constitutes the core theoretical contribution of this paper.

1. Information usage transparency in privacy policies has a double-edged effect on users’ continuance intention to use the app. High information usage transparency differs fundamentally from low information usage transparency. On one hand, high transparency reduces information asymmetry between service platforms and users. On the other hand, abundant, complex text content increases users’ cognitive load and leads to adverse outcomes. Two opposite influences on continuance intention emerge from this dual mechanism. The competing dual paths do not maintain identical intensity. Their effect sizes vary regularly with the sensitivity of the personal information involved. The difference forms distinct boundary conditions for the double-edged sword effect.
2. Perceived privacy control and perceived privacy fatigue both mediate the relationship between information usage transparency and users’ continuance intention to use the app. Full disclosure of rules for information collection and use improves transparency in information use. High transparency strengthens users’ sense of mastery over personal data and elevates perceived privacy control. At the same time, long, complex policy clauses increase reading difficulty and psychological pressure. The pressure further intensifies perceived privacy fatigue.
3. Supplementary analytical results confirm the differentiated moderating role of text information sensitivity. The variable adjusts the predictive links between information usage transparency and users’ privacy perceptions. Under high text information sensitivity, high information usage transparency has a stronger positive influence on perceived privacy control. Its promotional effect on perceived privacy fatigue weakens. Under low text information sensitivity, high information usage transparency yields only limited improvement in perceived privacy control. It exerts a more obvious boosting effect on perceived privacy fatigue. The heterogeneous moderating pattern yields varying strengths of the double-edged sword effect across scenarios. When users handle highly sensitive personal information, the positive path via perceived privacy control becomes dominant. The negative path driven by perceived privacy fatigue is weakened. When users face low-sensitivity information, the negative fatigue path stands out. The positive gain brought by perceived privacy control declines. This discrepancy across different scenarios forms the key innovative finding regarding the double-edged sword effect in this research.
4. The mediating effect of perceived privacy control is larger than that of perceived privacy fatigue. This outcome reflects users’ greater priority on control over their personal information than on the fatigue caused by complicated privacy policy texts. The moderating role of text information sensitivity works together with the two opposing mediating paths. The integrated results explain why the double-edged sword effect exhibits different intensities across varying privacy contexts.

### 5.2. Theoretical Contributions

First, this study enriches research on the mechanisms through which characteristics of privacy policy content influence users’ behavioral intentions. As a core tool for protecting user privacy, privacy policies play an increasingly important role in the digital economy. However, existing studies have reached inconsistent conclusions regarding how the content of privacy policies affects users’ continuance intention (Resnick & Montania, 2003; Srinath et al., 2021). Grounded in Stress and Coping Theory and Information Boundary Theory, this study adopts the user-perception perspective. It verifies that the transparency of privacy policy information produces a double-edged sword effect on users’ continuance intention. Specifically, this transparency enhances continuance intention by improving users’ perceived control over privacy. At the same time, it reduces continuance intention by exacerbating perceived privacy fatigue. This research addresses the limitation of prior studies, which examine only the single-dimensional effects of privacy policies. It establishes an in-depth link between the characteristics of privacy policy content and users’ actual usage behaviors. It also provides an integrated explanation for the contradictory effects of information-use transparency on user behavior. From the dual appraisal logic of Stress and Coping Theory, this study further delineates the theoretical divide between the benefit-enhancing path (perceived privacy control) and the detriment-weakening path (perceived privacy fatigue). Information usage transparency acts as a privacy stressor. Opportunity appraisal generates perceived control over privacy to boost continuous use. Threat appraisal triggers perceived privacy fatigue, thereby suppressing usage intention. These two opposing cognitive evaluations form competing parallel mediators. This resolves the ambiguity in the differentiation between dual mechanisms in the prior literature.

Crucially, this paper explains why perceived privacy control, rather than perceived trust, serves as the primary mediator. It also clarifies users’ cognitive distinction between the two constructs when they review privacy policies. First, their connotations differ fundamentally. Perceived trust refers to a broad affective belief in platform reliability. Perceived privacy control refers to users’ direct perception of their autonomy over the use of their personal data, which they derive from transparent clauses. Second, users form sequential judgments while reading policies. They first identify actionable data rights to perceive control. Trust only emerges as a secondary derivative feeling that arises after users perceive sufficient control. Third, perceived privacy control aligns better with Stress and Coping Theory. It reflects users’ self-perceived coping resources relative to privacy risks, which aligns with the theory’s core appraisal logic. Trust lacks this risk-coping cognitive attribute and cannot capture the proximal psychological effect of transparency. This distinction highlights the theoretical novelty of taking perceived privacy control as the core mediator.

Second, this study adopts perceived privacy fatigue and perceived privacy control as mediating variables. This approach unpacks the black box underlying the relationship between the transparency of privacy policies’ information usage and users’ continuance intention. Scholars have previously called for in-depth investigations into the mediating role of users’ psychological perceptions in privacy decision-making (Martin et al., 2017; Esteves et al., 2022). Nevertheless, most existing studies analyzed individual psychological variables separately. They failed to integrate the combined effects of multiple perceptions. According to Stress and Coping Theory, individuals develop dual cognitive evaluations of threat and opportunity when confronted with stressors. This study confirms that the content of privacy policies serves as an implicit stressor during app use. It triggers users’ dual psychological perceptions of opportunity and threat. This finding is consistent with the view that users hold ambivalent psychological perceptions toward privacy management, as proposed by Choi et al. (2018).

Finally, this study clarifies the moderating effect of text information sensitivity on the relationship between information usage transparency and users’ privacy perceptions. It also extends relevant research on text information sensitivity. The impact of information usage transparency on users’ privacy perceptions varies with the sensitivity of the text information. This demonstrates the critical moderating role of this variable. The results suggest that classifying information sensitivity and optimizing the design of information usage transparency for different types of information can effectively amplify the positive effects and mitigate the negative effects of transparency. Furthermore, as a situational variable in privacy policy research, text information sensitivity involves classifying information based on various risk attributes. From the perspective of enterprise-controllable situational factors, this study identifies the boundary conditions for the double-edged sword effect of information usage transparency.

### 5.3. Managerial Implications

First, app providers should fully recognize the double-edged nature of the sword. They should formulate strategies for optimizing privacy policies. These strategies should balance the value of transparency with users’ perceived cognitive load. This study uncovers differentiated boundary effects of text information sensitivity. Based on these effects, developers can adopt layered privacy disclosure and just-in-time on-demand disclosure frameworks. These frameworks realize targeted transparency design for different types of user data. Information usage transparency can boost users’ continuance intention by enhancing perceived privacy control. It can also undermine it by exacerbating perceived privacy fatigue. Therefore, providers should avoid extreme approaches. On the one hand, they should define reasonable boundaries for information disclosure based on specific business scenarios. For example, e-commerce apps may focus on disclosing how shopping behavior data is used. They should refrain from presenting redundant information irrelevant to core services. On the other hand, a tiered disclosure design is recommended. The core content of privacy policies can be summarized to satisfy users’ demand for quick access to key information. Detailed clauses can be folded for users who need further reference. This layered structure aligns with differentiated design logic for sensitive and non-sensitive data. Non-sensitive information can be disclosed in full and in detail to consolidate users’ perceived privacy control. Sensitive data uses condensed, summarized text to reduce cognitive load. This approach balances information completeness and cognitive convenience. It alleviates perceived privacy fatigue caused by information overload.

Second, app providers should target users’ perceived privacy fatigue and perceived privacy control. They should develop tailored schemes to improve psychological perceptions. This addresses the underlying mechanism by which transparency affects user decisions. To reduce perceived privacy fatigue, providers can reduce users’ information-processing costs. They can replace plain text with visual and interactive designs for easier comprehension. They can also highlight key clauses to save users time during screening. Just-in-time disclosure can be deployed to complement layered disclosure. It avoids flooding users with all privacy rules at the first launch. Detailed explanations of sensitive data collection only pop up when relevant functions are triggered. This approach does not stack all clauses in the initial privacy notice. To strengthen perceived privacy control, it is essential to improve users’ sense of active control. A one-click setting function can be offered. It allows users to quickly enable or disable authorization for sensitive information.

Finally, app providers should take text information sensitivity as the guideline for differentiated design. They should optimize privacy policies from the perspective of controllable situational factors. This maximizes the positive effects of transparency. For highly sensitive information such as phone numbers and identity data, providers should enhance both transparency and user control. They should specify in detail how such information is utilized. They should also adopt pop-up windows for secondary confirmation before accessing the data. For low-sensitivity information, such as storage space permissions, transparency descriptions can be simplified. This differentiated matching of disclosure depth perfectly resolves the double-edged dilemma of transparency. Sufficiently detailed transparency is provided for non-sensitive data without causing excessive fatigue. Concise, summarized disclosure is applied to sensitive data to retain transparency and curb cognitive burden. For instance, pop-up reminders can be displayed only when image saving is required. This avoids including excessive descriptions in the initial privacy policy that distract users. Furthermore, strategies can be adjusted by app category. Targeted design based on varying information types and usage scenarios helps mitigate the negative interference of text information sensitivity on transparency effects. It ultimately improves users’ acceptance and trust in privacy policies.

### 5.4. Limitations and Future Research

This study verifies the dual mechanisms by which information usage transparency influences users’ continuance intention. However, several limitations remain. Future research can be further conducted from the following two perspectives.

First, this study uses a scenario-based experimental design. This design may bias participants’ perceptions of information usage transparency. The bias is due to artificial contextual settings. Mixed-method research designs are recommended for future work. These designs can observe natural user attitudes toward real privacy policies. During stimulus development, independent reviewers refined the wording. They aimed to balance reading difficulty across text information sensitivity conditions. However, formal quantitative readability calibration (e.g., Flesch–Kincaid scores) was not implemented. Detailed depictions of sensitive data may independently trigger cognitive strain and perceived privacy fatigue. This offers an alternative explanation for the observed moderation effect. Future research should incorporate standardized readability evaluation in stimulus pretesting. This would isolate the true effect of information sensitivity. It would also remove cognitive load confounds.

Second, users’ continuance intention is influenced by various factors. This study only focuses on the mechanisms and boundary conditions. These mechanisms link information usage transparency to perceived privacy fatigue and perceived privacy control. This leaves room for further exploration. Future studies may incorporate user characteristics and additional privacy policy attributes. User characteristics include usage motivation, user type, and attribution of privacy policy motives. Additional attributes include optionality. These studies can further analyze the moderating mechanisms and boundary conditions. These conditions exist in the relationship between information usage transparency and users’ continuance intention.

Third, the dependent variables in this study did not fully satisfy the normality assumption required for parametric ANOVA. To address this issue, we used nonparametric tests (Mann–Whitney U and Scheirer–Ray–Hare) as the primary analytical framework, with parametric ANOVA serving only as cross-validation. This conservative approach reduces the risk of biased inferences. Future studies may consider larger sample sizes or alternative robust methods to further validate the stability of our findings.

## Figures and Tables

**Table 1:** Demographic information of the participants (*N* = 39).

Demographics	Category	Count	Rate (%)
Gender	Male	19	48.70
	Female	20	51.30
Age	Under 20 years old	23	59.00
	21–30 years old	16	41.00
Education	Bachelor’s degree	28	71.80
	Master’s degree	8	20.50
	Doctoral degree	3	7.70

**Table 2:** Results of Mann–Whitney U tests (*N* = 39).

Construct	Low Group (*N* = 17)	High Group (*N* = 22)	*Z*	*p*
Information Usage Transparency	2.50 (2.00, 2.75)	5.13 (3.75, 6.31)	−4.26	0.000 ***

Note: *** = *p* < 0.001.

**Table 3:** Demographic information of the participants (*N* = 30).

Demographics	Category	Count	Rate (%)
Gender	Male	16	53.30
	Female	14	46.70
Age	Under 20 years old	18	60.00
	21–30 years old	12	40.00
Education	Bachelor’s degree	19	63.30
	Master’s degree	10	33.30
	Doctoral degree	1	3.30

**Table 4:** Results of Mann–Whitney U tests (*N* = 30).

Construct	Low Group (*N* = 15)	High Group (*N* = 15)	*Z*	*p*
Text Information Sensitivity	2.33 (2.00, 3.00)	5.33 (4.00, 6.00)	−4.21	0.000 ***

Note: *** = *p* < 0.001.

**Table 5:** Demographic information of the participants (Experiment 1, *N* = 147).

Demographics	Category	Count	Rate (%)
Gender	Male	69	46.90
	Female	78	53.10
Age	Under 20 years old	38	25.90
	21–30 years old	83	56.50
	31–40 years old	26	17.70
Education	Below Bachelor’s degree	27	18.40
	Bachelor’s degree	71	48.30
	Master’s degree	38	25.90
	Doctoral degree	11	7.50

**Table 6:** Reliability and validity analysis (Experiment 1, *N* = 147).

Construct	Items	Factor Loading	AVE	CR	Cronbach’s Alpha
Perceived Privacy Control (PPC)	PPC1	0.748	0.616	0.865	0.864
	PPC2	0.851			
	PPC3	0.726			
	PPC4	0.809			
Perceived Privacy Fatigue (PPF)	PPF1	0.820	0.703	0.876	0.873
	PPF2	0.891			
	PPF3	0.801			
Continuous Use Intention (CUI)	CUI1	0.782	0.634	0.839	0.838
	CUI2	0.818			
	CUI3	0.788			

**Table 7:** Fornell–Larcker discriminant validity (Experiment 1, *N* = 147).

Variable	PPC	PPF	CUI
PPC	**0.785**		
PPF	−0.318 **	**0.839**	
CUI	0.651 **	−0.425 **	**0.796**

Note: ** *p* < 0.01, two-tailed test. Bold values on the diagonal represent the square root of each construct’s AVE. Off-diagonal entries are Pearson correlation coefficients between latent composite scores. PPC = perceived privacy control; PPF = perceived privacy fatigue; CUI = continuous use intention.

**Table 8:** Descriptive statistics and full-variable Pearson correlations (Experiment 1, *N* = 147).

Variable	M	SD	Gender	Age	Education	IUT	PPC	PPF	CUI
Gender	1.530	0.501	1						
Age	1.920	0.657	0.028	1					
Education	2.220	0.834	−0.041	0.296 **	1				
IUT	1.480	0.501	0.036	−0.087	−0.064	1			
PPC	4.255	1.261	0.069	−0.154	0.025	0.232 **	1		
PPF	4.408	1.294	−0.122	0.018	−0.100	0.211 *	−0.318 **	1	
CUI	4.002	1.188	0.067	−0.076	0.039	−0.002	0.651 **	−0.425 **	1

Note: * *p* < 0.05, ** *p* < 0.01, two-tailed test. IUT = information usage transparency (dummy variables); PPC = perceived privacy control; PPF = perceived privacy fatigue; CUI = continuous use intention.

**Table 9:** Results of Mann–Whitney U tests (Experiment 1, *N* = 147).

Construct	Low Group (*N* = 76)	High Group (*N* = 71)	*Z*	*p*
Perceived Privacy Control (PPC)	3.75 (3.25, 4.69)	4.75 (4.00, 5.50)	−3.28	0.001 **
Perceived Privacy Fatigue (PPF)	4.33 (3.33, 5.00)	4.67 (3.67, 5.67)	−2.40	0.016 *

Note: ** = *p* < 0.01; * = *p* < 0.05.

**Table 10:** Regression analysis results (Experiment 1, *N* = 147).

Variables	M1	M2	M3	M4
Constant	3.86 ***	5.96 ***	1.19 **	2.82 ***
	(8.24)	(10.81)	(2.66)	(4.95)
Gender	0.17	0.04	0.05	−0.02
	(0.88)	(0.21)	(0.35)	(−0.10)
Age	−0.18	−0.13	0.03	0.04
	(−1.14)	(−0.93)	(0.28)	(0.31)
Education Level	0.10	0.02	0.03	−0.01
	(0.82)	(0.20)	(0.26)	(−0.14)
Perceived Privacy Fatigue		−0.41 ***		0.54 ***
		(−5.90)		(8.83)
Perceived Privacy Control			0.61 ***	−0.25 ***
			(10.065)	(−4.27)
Sample Size	147	147	147	147
R-Squared	0.02	0.21	0.43	0.49
Adjusted R-Squared	−0.006	0.19	0.41	0.47
F-Value	0.73	9.39 ***	26.26 ***	27.19 ***

Note: ** = *p* < 0.01; *** = *p* < 0.001.

**Table 11:** Mediation effect test (Experiment 1, *N* = 147).

Mediation Path	Effect Size	Standard Error	95%CI	
			LLCI	ULCI
C1: Information Usage Transparency → Perceived Privacy Fatigue → Continuance Intention	−0.13	0.07	−0.28	−0.02
C2: Information Usage Transparency → Perceived Privacy Control → Continuance Intention	0.31	0.12	0.08	0.57
C2-C1	0.44	0.13	0.19	0.68

**Table 12:** Demographic information of the respondents (Experiment 2, *N* = 182).

Demographics	Category	Count	Rate (%)
Gender	Male	95	52.20
	Female	87	47.80
Age	Under 20 years old	51	28.00
	21–30 years old	91	50.00
	31–40 years old	30	16.50
	41 years old or above	10	5.50
Education	Below Bachelor’s degree	43	23.60
	Bachelor’s degree	98	53.80
	Master’s degree	33	18.10
	Doctoral degree	8	4.40

**Table 13:** Reliability and validity analysis (Experiment 2, *N* = 182).

Construct	Items	Factor Loading	AVE	CR	Cronbach’s Alpha
Perceived Privacy Control (PPC)	PPC1	0.866	0.683	0.896	0.896
	PPC2	0.809			
	PPC3	0.819			
	PPC4	0.811			
Perceived Privacy Fatigue (PPF)	PPF1	0.825	0.681	0.865	0.863
	PPF2	0.809			
	PPF3	0.841			
Continuous Use Intention (CUI)	CUI1	0.835	0.612	0.825	0.824
	CUI2	0.734			
	CUI3	0.775			

**Table 14:** Fornell–Larcker discriminant validity (Experiment 2, *N* = 182).

Variable	PPC	PPF	CUI
PPC	**0.826**		
PPF	−0.270 **	**0.825**	
CUI	0.482 **	−0.340 **	**0.782**

Note: ** *p* < 0.01, two-tailed test. Bold values on the diagonal represent the square root of each construct’s AVE. Off-diagonal entries are Pearson correlation coefficients between latent composite scores. PPC = perceived privacy control; PPF = perceived privacy fatigue; CUI = continuous use intention.

**Table 15:** Descriptive statistics and full-variable Pearson correlations (Experiment 2, *N* = 182).

Variable	M	SD	Gender	Age	Education	IUT	PPC	PPF	CUI
Gender	1.480	0.501	1						
Age	1.990	0.818	−0.088	1					
Education	2.030	0.772	0.131	0.018	1				
IUT	1.530	0.501	−0.086	−0.128	−0.060	1			
PPC	4.132	1.157	0.165 *	−0.093	0.012	0.256 **	1		
PPF	4.291	1.214	−0.185 *	0.054	0.027	0.219 **	−0.270 **	1	
CUI	3.808	1.165	0.086	−0.034	0.017	−0.034	0.482 **	−0.340 **	1

Note: * *p* < 0.05, ** *p* < 0.01, two-tailed test. IUT = Information Usage Transparency (dummy variables); PPC = perceived privacy control; PPF = perceived privacy fatigue; CUI = continuous use intention.

**Table 16:** Results of Mann–Whitney U tests (Experiment 2, *N* = 182).

Construct	Low Group (*N* = 86)	High Group (*N* = 96)	*Z*	*p*
Perceived Privacy Control (PPC)	3.75 (3.25, 4.50)	4.50 (3.75, 5.25)	−3.92	0.000 ***
Perceived Privacy Fatigue (PPF)	4.00 (3.33, 4.67)	4.67 (3.67, 5.67)	−2.75	0.006 **

Note: *** = *p* < 0.001; ** = *p* < 0.01.

**Table 17:** Regression analysis results (Experiment 2, *N* = 182).

Variables	M1	M2	M3	M4
Constant	3.58 ***	5.09 ***	1.72 ***	2.98 ***
	(8.81)	(10.13)	(3.89)	(5.29)
Gender	0.19	0.05	0.01	−0.07
	(1.09)	(0.26)	(0.09)	(−0.43)
Age	−0.04	−0.02	0.02	0.02
	(−0.36)	(−0.21)	(0.17)	(0.23)
Education Level	0.01	0.04	0.02	0.03
	(0.09)	(0.34)	(0.16)	(0.34)
Perceived Privacy Fatigue		−0.32 ***		−0.22 ***
		(−4.67)		(−3.45)
Perceived Privacy Control			0.49 ***	0.43 ***
			(7.19)	(6.34)
Sample Size	182	182	182	182
R-Squared	0.008	0.12	0.23	0.28
Adjusted R-Squared	−0.009	0.10	0.22	0.26
F-Value	0.49	5.86 ***	13.39 ***	13.76 ***

Note: *** = *p* < 0.001.

**Table 18:** Mediation effect test (Experiment 2, *N* = 182).

Mediation Path	Effect Size	Standard Error	95%CI	
			LLCI	ULCI
C1: Information Usage Transparency → Perceived Privacy Fatigue → Continuance Intention	−0.09	0.05	−0.21	−0.01
C2: Information Usage Transparency → Perceived Privacy Control → Continuance Intention	0.25	0.08	0.11	0.42
C2-C1	0.33	0.08	0.19	0.50

**Table 19:** Demographic information of the respondents (Experiment 3, *N* = 200).

Demographics	Category	Count	Rate (%)
Gender	Male	101	50.50
	Female	99	49.50
Age	Under 20 years old	57	28.50
	21–30 years old	98	49.00
	31–40 years old	28	14.00
	41 years old or above	17	8.50
Education	Below Bachelor’s degree	37	18.50
	Bachelor’s degree	112	56.00
	Master’s degree	42	21.00
	Doctoral degree	9	4.50

**Table 20:** Reliability and validity analysis (Experiment 3, *N* = 200).

Construct	Items	Factor Loading	AVE	CR	Cronbach’s Alpha
Perceived Privacy Control (PPC)	PPC1	0.852	0.757	0.926	0.925
	PPC2	0.864			
	PPC3	0.886			
	PPC4	0.877			
Perceived Privacy Fatigue (PPF)	PPF1	0.794	0.595	0.815	0.815
	PPF2	0.788			
	PPF3	0.731			
Continuous Use Intention (CUI)	CUI1	0.826	0.711	0.880	0.880
	CUI2	0.871			
	CUI3	0.831			

**Table 21:** Fornell–Larcker discriminant validity (Experiment 3, *N* = 200).

Variable	PPC	PPF	CUI
PPC	**0.870**		
PPF	0.204 **	**0.771**	
CUI	0.409 **	−0.346 **	**0.843**

Note: ** *p* < 0.01, two-tailed test. Bold values on the diagonal represent the square root of each construct’s AVE. Off-diagonal entries are Pearson correlation coefficients between latent composite scores. PPC = perceived privacy control; PPF = perceived privacy fatigue; CUI = continuous use intention.

**Table 22:** Descriptive statistics and full-variable Pearson correlations (Experiment 3, *N* = 200).

Variable	M	SD	Gender	Age	Education	IUT	TIS	PPC	PPF	CUI
Gender	1.500	0.501	1							
Age	2.030	0.876	−0.154 *	1						
Education	2.120	0.751	0.035	0.050	1					
IUT	1.500	0.501	−0.010	0.006	0.007	1				
TIS	1.500	0.501	0.050	0.029	0.087	0.000	1			
PPC	4.421	1.362	0.006	0.047	0.059	0.242 **	0.150 *	1		
PPF	4.370	1.170	−0.082	−0.068	−0.092	0.194 **	−0.146 *	0.204 **	1	
CUI	3.447	1.515	0.074	0.032	0.003	0.174 *	0.051	0.409 **	−0.346 **	1

Note: * *p* < 0.05, ** *p* < 0.01, two-tailed test. IUT = information usage transparency (dummy variables); TIS = text information sensitivity (dummy variables,); PPC = perceived privacy control; PPF = perceived privacy fatigue; CUI = continuous use intention.

**Table 23:** Demographic information of the respondents (*N* = 44).

Demographics	Category	Count	Rate (%)
Gender	Male	24	54.50
	Female	20	45.50
Age	Under 20 years old	16	36.40
	21–30 years old	20	45.50
	31–40 years old	6	13.60
	41 years old or above	2	4.50
Education	Below Bachelor’s degree	10	22.70
	Bachelor’s degree	21	47.70
	Master’s degree	10	22.70
	Doctoral degree	3	6.80

**Table 24:** Results of Mann–Whitney U tests (*N* = 44).

Construct	Low Group (*N* = 22)	High Group (*N* = 22)	*Z*	*p*
Information Usage Transparency	2.38 (1.75, 2.81)	5.38 (4.25, 6.06)	−5.19	0.000 ***
Text Information Sensitivity	4.00 (3.00, 5.42)	3.67 (2.33, 5.50)	−4.92	0.000 ***

Note: *** = *p* < 0.001.

**Table 25:** Results of Mann–Whitney U tests (Experiment 3, *N* = 200).

Construct	Low Group (*N* = 100)	High Group (*N* = 100)	*Z*	*p*
Perceived Privacy Control (PPC)	4.25 (3.25, 5.00)	4.75 (3.31, 6.25)	−3.33	0.001 **
Perceived Privacy Fatigue (PPF)	4.33 (3.00, 5.33)	4.33 (3.75, 5.67)	−2.44	0.015 *

Note: ** = *p* < 0.01; * = *p* < 0.05.

**Table 26:** Regression analysis results (Experiment 3, *N* = 200).

Variables	M1	M2	M3	M4
Constant	3.92 ***	6.25 ***	2.11 ***	4.76 ***
	(7.15)	(9.04)	(3.64)	(7.75)
Gender	0.24	0.15	0.23	0.10
	(1.11)	(0.71)	(1.15)	(0.56)
Age	0.08	0.03	0.04	−0.04
	(0.62)	(0.21)	(0.35)	(−0.37)
Education Level	0.005	−0.06	−0.05	−0.14
	(0.04)	(−0.44)	(−0.34)	(−1.23)
Perceived Privacy Fatigue		−0.45 ***		−0.59 ***
		(−5.07)		(−7.68)
Perceived Privacy Control			0.46 ***	0.56 ***
			(6.26)	(8.63)
Sample Size	200	200	200	200
R-Squared	0.007	0.12	0.17	0.37
Adjusted R-Squared	−0.008	0.11	0.16	0.35
F-Value	0.48	6.83	10.24 ***	22.41 ***

Note: *** = *p* < 0.001.

**Table 27:** Mediation effect test (Experiment 3, *N* = 200).

Mediation Path	Effect Size	Standard Error	95%CI	
			LLCI	ULCI
C1: Information Usage Transparency → Perceived Privacy Fatigue → Continuance Intention	−0.28	0.11	−0.51	−0.09
C2: Information Usage Transparency → Perceived Privacy Control → Continuance Intention	0.35	0.10	0.15	0.56
C2-C1	0.63	0.16	0.31	0.97

**Table 28:** Moderated mediation effect test (Experiment 3, *N* = 200).

Moderator	Effect	BootSE	BootLLCI	BootULCI
**Information Usage Transparency → Perceived Privacy Fatigue → Continuance Intention**				
Low Text Information Sensitivity	−0.49	0.18	−0.86	−0.17
High Text Information Sensitivity	−0.07	0.12	−0.32	0.18
Difference Index	0.42	0.22	0.02	0.86
**Information Usage Transparency → Perceived Privacy Control → Continuance Intention**				
Low Text Information Sensitivity	0.12	0.14	−0.16	0.40
High Text Information Sensitivity	0.57	0.15	0.29	0.89
Difference Index	0.45	0.21	0.05	0.89

## Data Availability

The raw data supporting the conclusions of this article will be made available by the authors on request.

## Abbreviations

The following abbreviations are used in this manuscript:

IUT	Information usage transparency
TIS	Text information sensitivity
PPC	Perceived privacy control
PPF	Perceived privacy fatigue
CUI	Continuous use intention

## Appendix A

**Table A1:** Measurement items.

Construct	Items
Perceived Privacy Control (PPC)	1. I believe I can take control over my personal information.
	2. I can decide the extent to which this mobile app uses my personal information.
	3. I have a say in how this mobile app utilizes my personal information.
	4. I have the right to decide whether my personal information can be shared with third parties.
Perceived Privacy Fatigue (PPF)	1. I am often distracted by excessive information when making privacy-related decisions on this app.
	2. I feel overwhelmed by the large volume of information I need to process every day.
	3. There is far too much information presented on this mobile app rather than an appropriate amount.
Continuous Use Intention (CUI)	1. I intend to keep using this mobile app instead of discontinuing it.
	2. I intend to continue using this mobile app rather than switching to alternative platforms.
	3. I would stop using this mobile app if I had the chance.

**Table A2:** Normality and homogeneity of variance test (Experiment 1, *N* = 147).

Variable	Group	Kolmogorov–Smirnov (K-S)		Levene’s Test for Homogeneity of Variance			
		Statistic	*p*	Statistic	df1	df2	*p*
Perceived Privacy Control (PPC)	Low IUT	0.121	0.008	0.769	1	145	0.382
	High IUT	0.097	0.094				
Perceived Privacy Fatigue (PPF)	Low IUT	0.122	0.007	3.225	1	145	0.075
	High IUT	0.101	0.072				

**Table A3:** Normality and homogeneity of variance test (Experiment 2, *N* = 182).

Variable	Group	Kolmogorov–Smirnov (K-S)		Levene’s Test for Homogeneity of Variance			
		Statistic	*p*	Statistic	df1	df2	*p*
Perceived Privacy Control (PPC)	Low IUT	0.086	0.171	1.721	1	180	0.191
	High IUT	0.123	0.001				
Perceived Privacy Fatigue (PPF)	Low IUT	0.147	0.000	3.641	1	180	0.058
	High IUT	0.107	0.009				

**Table A4:** Normality and homogeneity of variance test (Experiment 3, *N* = 200).

Variable	Group	Kolmogorov–Smirnov (K-S)		Levene’s Test for Homogeneity of Variance			
		Statistic	*p*	Statistic	df1	df2	*p*
Perceived Privacy Control (PPC)	Low IUT	0.137	0.000	20.306	1	198	0.000
	High IUT	0.134	0.000				
Perceived Privacy Fatigue (PPF)	Low IUT	0.142	0.000	1.033	1	198	0.311
	High IUT	0.124	0.001				
